# The Integrated Policy Package Assessment approach: elaborating ex ante knowledge in the field of urban mobility

**DOI:** 10.1186/s13705-022-00362-4

**Published:** 2022-09-07

**Authors:** Dirk Scheer, Marion Dreyer, Maike Schmidt, Lisa Schmieder, Annika Arnold

**Affiliations:** 1 Institute for Technology Assessment and Systems Analysis (ITAS), Karlsruhe Institute of Technology (KIT), Karlsruhe, Germany; 2 DIALOGIK Non-profit Institute for Communication and Cooperation Research, Stuttgart, Germany; 3 Center for Solar Energy and Hydrogen Research Baden-Württemberg (ZSW), Stuttgart, Germany; 4 Stuttgart Research Center for Interdisciplinary Risk and Innovation Studies (ZIRIUS), University of Stuttgart, Stuttgart, Germany

**Keywords:** Energy transition, Policy packages, Impact assessment

## Abstract

**Background:**

In response to climate change challenges, a main policy emphasis is on transitioning the energy system from high- to low-carbon energy supply. The German energy transition is first and foremost based on political decisions and interventions. These decisions need to be assessed ex ante to ensure a good governance approach to energy policies, for which this paper introduces the Integrated Policy Package Assessment approach (IPPA). IPPA consists of four steps: design, assessment, evaluation and discourse.

**Results:**

The results section illustrates the IPPA framework by applying it to urban passenger transport as an example case. First, the design phase was used to elaborate two complementary policy packages each consisting of several policy measures in the transformation pathways of “multi- and inter-modality”, and “alternative drive”. Second, the individual measures of the packages were impact-analysed by a large number of individual impact studies from various disciplines. Synthesizing the individual study results, we developed an impact assessment matrix for impact evaluation. The matrix covers the impact categories: technology development, sector integration, environment, social resonance, and institutional factors. In a further step, the key findings of the impact assessment were reflected and reviewed from the perspectives of various stakeholders and practice experts through a practice–science dialogue on transforming the urban passenger transport system.

**Conclusions:**

The discussion and conclusion sections outline the main findings relating to content and process aspects, when applying the IPPA framework to a policy package in urban transport.

## Background

The transformation of the energy system towards climate compatibility and sustainability—commonly referred to in Germany as ‘the energy turnaround’—is a fundamental process of socio-technical change, which must be actively and purposefully shaped as a “joint effort” [1]. The socio-technical transformation process is characterized by complexity, uncertainty and ambiguity [2–5]. The high degree of complexity results from the systemic intertwining of infrastructure, technology, behaviour, market design and politics. There is great uncertainty with regard to technical development, the decisions of actors and their interactions, or overall future developments both within and outside the energy system. Ambiguity refers to the variety of preferences of citizens, stakeholders, entrepreneurs and decision-makers as to the path to be taken for energy system transformation.

The energy system is widely understood as a highly interconnected socio-technical system with both sector-specific and cross-sectoral system characteristics and rationalities [6–8]. Throughout the supply, distribution and use of energy in the electricity, heat and mobility sectors, technical components are closely linked to social and institutional actors and their individual and collective decisions. In such an understanding, technical, institutional, economic and social parameters come together and interact closely. The energy system as it is today or is desired to be in the future is thus a manifestation of this interplay and is characterized by a high degree of complexity [9]. Regarding knowledge of energy supplies, it is a challenge to determine the exact initial configuration (boundary conditions) and the interactions between the influencing factors (interdependencies) intersubjectively [10].

The transport sector is an excellent example of such complexity, uncertainty and ambiguity [11, 12]. The sector is much more complex than other energy sectors. Despite all the political objectives, it has not yet been possible to reduce greenhouse gas emissions from transport in Germany decisively below the 1990 level. On the contrary, emissions are rising continuously—even if a slight decrease can be observed in 2018 [13]. Economic interdependencies in highly specialized value chains with a demand for highly qualified, mobile workers with mobility seen as an expression of freedom, individuality and independence, create tension in discussions on the “mobility transition” [14]. Several objectives are linked to the transformation of mobility systems, covering a much broader range of issues than just climate protection through greenhouse gas reduction [15]. The aim is to improve quality of life by reducing air and noise pollution and by making streets, neighbourhoods and cities more human-oriented than car-focused, but also to save time by relieving the burdens on infrastructure and avoiding traffic jams. At the same time, it should be possible to satisfy the mobility needs of individuals. Further, today’s degree of mobility achieved should be maintained, if not even further increased. This is important, as mobility is equated with individual degrees of freedom which should not be curtailed.

Thus, an integrated approach is required. Our research has addressed the question of how to link sustainability target achievement with transition pathways through the ex ante assessment of policy impact, reflecting pros and cons with decision-makers and stakeholders. The Integrated Policy Package Assessment (IPPA) approach is a suggested way to meet these requirements. This paper elaborates its use to assess ex ante knowledge in the field of urban passenger transport.

The paper is organized as follows: “Methods” section sets out the aim and leading research question, and links the work with a literature review. In “Results” section, we present the main results, outlining the IPPA conceptual framework and the IPPA case study focusing on example of the “alternative drives”. Finally, “Discussion” section discusses the results, summarizes the focal points, and draws some conclusions.

## Methods

### Leading research question

A substantial social–technical process such as the energy and mobility transition raises fundamental questions. What are promising pathways towards the transition? Which policy interventions and packages are suitable and functionally equivalent? How can the effects and side-effects of interventions in complex policy areas be assessed? What consequences are to be expected from policies, for example, for the environment, taxpayers, companies or individual quality of life? And what are stakeholders’ opinions on proposed transition pathways, policies and their corresponding impacts?

The energy transition is first and foremost based on political decisions, since promising transformation paths towards a climate-compatible future can only be realized through political intervention and adequate policy action. In short: no decision—no transition. However, a key question for political decision-makers is which decisions should be taken to reach sustainability goals, considering both the intended and unintended effects against the background of alternative decisions which may reach the same transition target, but show different impact consequences. This is no easy task, since the assessment of future-oriented pathways is based on an array of hypothetical ex ante assumptions and uncertainties in the area of anticipating future knowledge for socio-technical systems.

What is needed to meet these requirements? The leading research question calls for an integrated approach that closely links the design of adequate transition policies and assessment of their impact profiles as ex ante knowledge provision for decision-makers. The IPPA approach is our contribution, and we illustrate it by applying it to an example case. The twofold task as presented in the “Results” section combines conceptual framework development with case study-oriented implementation work. In this paper, we thus present the IPPA approach, which aims to elaborate ex ante knowledge for experts and decision-makers based on inter- and trans-disciplinary research methods. As decision-makers are well aware of what positive and/or negative impacts policy measures will yield, the idea is to provide ex ante knowledge of future impacts on a multidimensional scope with regard to specific policies or policy packages. The overarching objective is to elaborate a framework that integrates heterogeneous research from different disciplines, synthesizes the results in a coherent evaluation matrix, which reflects the expectations and assessments of practitioners and stakeholders.^1^

### Literature review and methods

The IPPA approach as conceptualized and illustrated in this paper draws on several research topics in the field of social science-based sustainability research, technology assessment, and system analysis. The conceptual steps of IPPA comprise the four phases of design, assessment, evaluation, and discourse. *Design* means combining several policy measures and interventions into a coherent policy package [20–23]. *Analysis* means an interdisciplinary impact assessment in which the various effects of policy packages are assessed ex ante from different disciplinary perspectives [24, 25]. *Evaluation* means a synthesis of assessment outputs [26, 27]. Finally, *discourse* comprises a dialogue-based exchange and review by practitioners and stakeholders [28–30]. To sum up, the methodological process specified the overarching objective of integrating and synthesizing heterogeneous impact assessment of policy measures, including the reflections of practice actors and stakeholders as joint application-oriented research. It thus draws on the areas of literature concerning policy packages, impact assessment, and research involving societal actors.

Research into *policy packages* relates to research regarding policymaking. Standard practice in policymaking is often called “policy patching” [31–33]. Thus, empirical research on the design and content of national and/or international energy and climate policies often shows an ad hoc policy patching pattern. This pattern implies uncontrolled growth of local, regional, and federal state policies and interventions that are rarely consistent with one other [34, 35]. The policy package approach tries to tackle this problem “by considering the interaction of the instruments in a bundle” [36], considering one or more core policy measure(s) in combination with ancillary measures. The ancillary measures need to have one of three rationales. They should either increase the effectiveness of the primary measure, strengthen the acceptance of the primary measure, or facilitate political support for the primary measure [37–39]. The result is a policy package that is, ideally, as effective, efficient, and accepted as possible in order to cope with a given problem [40]. The package mitigates possible unintended effects, increases legitimacy and acceptance of the measures, and/or facilitates their implementation [41]. The approach is usually applied to the design of theory-based policy packages in scientific and teaching environments outside the real-world policy arena [42]. Within this paper, we combined the policy package approach with sustainable pathway identification and policy impact assessment and evaluation.

Sustainability has become the keyword for future orientation to safeguarding societies worldwide in harmony with one another and the biosphere [43]. It is first and foremost a social construct that seeks to improve the quality of life for the world’s peoples [44]. A key issue of sustainability is to integrate the three dimensions of economic, environmental and social (including socio-political) wellbeing targets. *Sustainability research* serves these efforts in many ways across the three dimensions. Among others, sustainability research documents the status quo, extrapolates and forecasts future developments, develops coherent sustainability transformation pathways, provides problem-solving metrics, indicators and tool assessments, addresses ethics, conflicting goals and deliberation processes, and helps to develop branch and sector-oriented sustainability specifications [45–50]. A special focus within sustainability research is on integrated impact assessment and evaluation, and sustainability target orientation. There is, for instance, a vast body of literature on sustainability impact assessment for the field energy transition and/or energy technologies [51–54]. Within the IPPA framework, we tackle several relevant issues of sustainability research, namely the field of sustainable pathway identification and interdisciplinary ex ante impact assessment. The concept of policy packaging should not focus only on the expected direct impact of the suggested measures, but has to foresee unexpected side-effects and unintended interrelationships with other sustainability goals. Interdisciplinary impact assessment from a variety of science disciplines therefore provides a multidimensional assessment picture in the areas of economic, environmental, and social dimensions.

The field of *Responsible Innovation (RI) and Responsible Research and Innovation (RRI)* [55–58] address the question of the responsible design and governance of research and innovation processes. The underlying idea is to steer the research and innovation process towards societally beneficial objectives. Science and research on RI/RRI were initially focused on technologies and processes with great societal transformation potential [59] as well as considerable scientific and ethical uncertainties [60]. A distinctive feature of RRI as understood in large parts of the literature [61, 62] is that steering innovation processes according to societal values and needs is interpreted as a collective responsibility. In this view, there is not only an obligation (for example for technology developers or policymakers) to organize inclusive and participatory processes. There is also an obligation for societal actors to engage in a collective debate that shapes the context for collective decision-making. It is in this regard that IPPA can be described as overlapping with RI/RRI approaches. The overlap is mainly in the dimensions of ‘anticipation’ and ‘inclusion’. The ‘evaluation’ component of the IPPA approach includes the different policy measures (or innovations in terms of new technology, infrastructure, policy, regulation) which compose the policy packages, and their interactions, in an ex ante impact analysis informed by the results of a preceding interdisciplinary analysis. This corresponds with RI/RRI’s dimension of ‘anticipation’ [63]. As participative approaches, we used different methods such as stocktaking workshops, a Group Delphi workshop, and a Practice-Science Dialogue.

## Results

### The IPPA framework concept

**Fig. 1:**
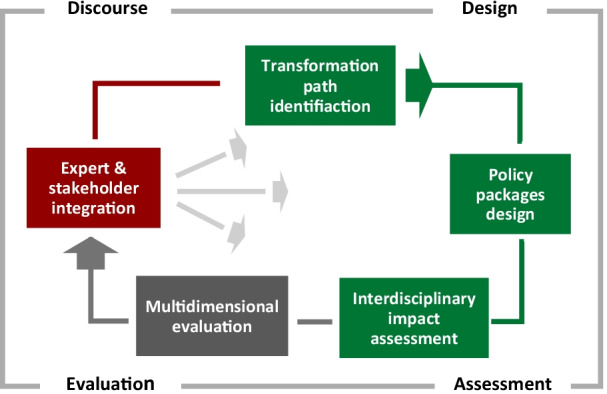
The ideal-type Integrated Policy Package Assessment approach (source: own elaboration based on [64])

The main intention of the IPPA approach was to develop an assessment procedure that integrated evidence and knowledge on functionally equivalent policy packages which aim to implement sustainable pathways towards climate-friendliness, whilst taking inter- and trans-disciplinary principles into account. As a result, we developed an IPPA framework concept consisting of a four-step phase-model with design, assessment, evaluation and discourse as its constituent elements (cf. Fig. 1).

At the heart of the approach is the *design of policy packages* that are capable of triggering the two transformation paths of “multi- and inter-modality” and “alternative drive systems”, here within the case study of urban passenger transport. The policy packages were developed using a mixed-method design consisting of a literature review, a participatory Group Delphi workshop, and a practice actors’ feedback workshop. In this paper, we will illustrate IPPA implementation according to the “alternative drive” case. The *assessment* of the policy packages comprised individual impact studies elaborated by contributions from ENavi project research groups. These were equally important, since each study’s method is characterized by specific strengths and weaknesses, and only their combination leads to robust results. The *evaluation* integrated and synthesized the individual impact studies into a coherent evaluation matrix. The integration aimed to deliver key insights on progress towards the mobility transition (intended impacts), and unintended side-effects and negative consequences. Finally, in the *discourse* phase, we applied several participatory methods linking discourse with the other framework phases. The policy package design, for instance, processed a Group Delphi workshop evaluating promising pathways and policy interventions, while the impact profiles of the policy packages were subjected to discussion and review by members of “competence teams” and further practice actors. The competence teams were a structural element of the ENavi project which included individuals from the economic and service sector, civil society, and administration concerned with issues around electricity, heat and/or mobility.

### The IPPACase study implementation for urban passenger transport

#### The design phase: developing the “alternative drive” policy package

The policy package design started with an extensive literature review in order to frame and specify promising transition pathways towards a sustainable urban mobility transition in 2050. As a result, two sustainable, complementary pathways seen as an integrated push- and pull approach were identified:

- the multi- and inter-modality pathway, and
- the alternative drive pathway.

For each pathway, we developed a pathway target definition and compiled adequate policies for pathway implementation via policy packages. The complementary relation of both pathways envisages first, a modal shift towards sustainable effective and efficient transport systems, and second a substitution towards alternative drives in the remaining vehicles. In this paper, we limit the presentation to the “alternative drive” pathways due to a lack of space. The results of the “multi- and inter-modality pathway” will be published separately. For the “alternative drive” pathway, the target formulation reads as follows:

The transformation path “alternative drives” contributes substantially to a climate-friendly and sustainable transport system in the remaining motorized private transport (MPT) in 2050. With the focus on changes on the supply side due to technology development, the penetration of the vehicle fleet with highly efficient alternative drive technologies supplemented by the provision of a climate-neutral fuel mix is centre stage. Alternative drive refers to the diffusion of purely battery electric vehicles, hybrid vehicles and fuel cell vehicles as well as further efficiency improvements in conventional drives powered by climate-neutral liquid and gaseous fuels (first- and second-generation biofuels, biomass to liquids, power to liquids, power to gas), which also contributes to a climate-neutral MPT. The technology mix of the vehicle fleet and the fuel consumption in 2050 will differ considerably from the year 2021.

The target formulation has been underpinned by desk research analysing several features of a future sustainable urban mobility. The background analysis identified cause–impact chains, indicators, scope, and target states of a sustainable urban mobility “vision”. The *policy package of alternative drives* resulted in two core measures accompanied by four ancillary measurements. Table 1 gives an overview of the measures and details the policies. In the following, we summarize the single measures.

**Table 1:** Policy Package “Increase of alternative drive via CO_2_ emission performance standards, and a CO_2_ price component for fossils fuels” (“alternative drives”)

*1. Core: “CO*_*2*_* emission performance standards of 60 g/km by 2030”*
• What: Setting a fleet limit for newly registered vehicles in Europe (starting value 95 g/km in 2020, reduction to 60 g/km by 2030); closing the current gap between the standard value (NEDC) and the real value of about 40% • Objective: To increase the supply of vehicles with alternative drive systems • Type: regulatory
*2. Core: “CO*_*2*_* price component for fossil fuels”*
• What: Introduction of a CO_2_ price component for fossil fuels that ensures the mathematically necessary increase in user costs of 2% p.a. from 2010 to 2030. For petrol, a total sum of 83.7 ct/L from the mineral oil tax and the CO_2_ component must be achieved in 2030, for diesel a total of 89.2 ct/L. Setting the CO_2_ price component at €150 per t of CO_2_ in 2030 (equivalent to 36.7 ct/L of petrol and 39.6 ct/L of diesel). At the same time adjustment of the mineral oil tax: for petrol, reduction from today’s 66.96 ct/L to 47 ct/L; for diesel, increase from today’s 46.38 ct/L to 49.6 ct/L • Objective: To reduce the attractiveness of conventionally operated vehicles by increasing variable costs (costs of use) by 2% p.a. (2010 to 2030) • Type: economic
*3. Ancillary: “Reform of the motor vehicle tax”*
• What: Conversion of the motor vehicle tax to CO_2_ emissions as the sole assessment variable. The tax exemption for electric vehicles remains in place. Up to a limit of 95 g, 0.40 euros per gram will be charged, and from 96 to 115 g/km 0.80 euros per gram. Between 115 and 135 g/km, 2.00 euros per gram will be charged, and over 136 g/km 5.00 euros/g will be charged. Above 200 g/km the amount per gram of CO_2_ rises to 15.00 Euro • Objective: To further reduce the attractiveness of conventionally powered vehicles by increasing the fixed costs (maintenance costs) for vehicles with fossil-fuelled drives • Type: economic
*4. Ancillary: “Technology development for intelligent charging & tariff systems”*
• What: The on-going development and expansion of the public charging infrastructure (= charging points in public spaces that can be provided by public or private providers) will be continued and supplemented by encouraged technology development which aims for solutions for intelligent, network-related charging considering, e.g. adequate tariff systems • Goal: Unproblematic integration of charging processes into the electricity system, avoidance of user restrictions • Type: promotional policy
*5. Ancillary: “Guideline on parking fees”*
• What: The objective of this measure is the gradual and transparently announced increase in parking costs by 50% by 2030 compared to the current level. Since the municipalities have to implement this step, a guideline for municipalities on the climate-friendly design of parking fees is to be drawn up as part of this measure. The municipalities should then adapt their parking fee structures accordingly • Objective: In order to reduce the attractiveness of conventional vehicles, the aim is to make parking more expensive, preferably for conventional vehicles • Type: informative
*6. Ancillary: “Target group-oriented information campaign on electric mobility”*
• What: A target group-oriented information campaign is to be developed and launched to help overcome the reluctance and scepticism towards electric mobility. The target groups should be private users as well as commercial and fleet operators • Objective: Closing knowledge gaps, reducing risk perception, supporting the purchase decision • Type: informative

Source: own elaboration

The core measure “CO_2_ fleet limit value of 60 g/km by 2030” is a lead instrument from the field of the regulatory toolbox. The CO_2_ limit for new cars is considered to have the most far-reaching effect as it addresses the entire new car fleet and almost all manufacturers (with the exception of very small fleets). This measure is a typical technology push measure, as it forces manufacturers to push forward technological developments and offer them on the market, with a focus on CO_2_ emission reduction during the car usage phase.

In contrast, the core measure “introduction of a CO_2_ component for fossil fuels” is a clear pull measure from the field of economic instruments, which primarily aims to increase the user costs for the MPT based on conventional propulsion and fossil fuels. By privileging alternative drive systems, the measure is intended to create incentives for users to decide to purchase vehicles with alternative drive systems. This will be directly supported by the accompanying measures “reform of the motor vehicle tax” in favour of climate-friendly, fuel-efficient and light vehicles, and “guidelines on parking fees”.

Both measures are intended to further increase the attractiveness of alternative drive systems with additional economic advantages, and thus to encourage users to buy them. Both core measures are flanked by ancillary measures in the field of infrastructure and communication. By promoting “intelligent charging points and tariff systems”, the aim is to prepare for the widespread integration of electric vehicles into the electricity grid so that no new barriers to the diffusion of electric vehicles arise. In addition, a target group-specific “information campaign on electric mobility” aims to fill information gaps and provide neutral information on the entire spectrum of electric mobility. In addition, measures in place, such as the privileged treatment of electric vehicles under the German Electromobility Act (EmoG) or tax advantages for company cars need to be continued.

#### The assessment phase: processing interdisciplinary impact studies

The assessment phase was based on sub-projects carried out within the ENavi. These were partly initiated independently from the IPPA approach. In a screening phase, we identified and evaluated on-going ENavi research via written surveys among the project staff [65]. We identified 19 individual impact studies contributing to the alternative drive policy package assessment. Table 2 gives an overview of the impact study’s main objective, the method used and the scientific discipline assigned to the corresponding policy measures. The impact assessment involved various scientific disciplines (political science, institutional economics, industrial economics, innovation economics, macroeconomics, microeconomics, resource economics and environmental psychology). In addition, law, engineering sciences and simulation sciences with different model approaches were involved. Methodologically, the research included a variety of methods, including desk research, document analysis, legal analysis, expert interviews, surveys, conjoint analyses, and computer models. In Table 2, we indicate the availability of data and results.

**Table 2:** Overview of included impact studies

No. and study objective*	Method	Discipline
*Core I: “CO*_*2*_* emission performance standards of 60 g/km by 2030”*		
1. Impact of CO_2_ limit values on vehicle fleet composition [n.a.]	Cost calculation	Innovation economics
2. Macroeconomic impact of climate policy instruments (CO_2_ price, ETS) within different scenarios [66]	Simulation (general equilibrium)	Energy economics
3. Impact assessment of CO_2_ emission standards vs. CO_2_ price instrument comparison [n.a.]	Decision theory	Institutional economics
4. Analysis of raw material availability (rare earth) for electro mobility [67]	Simulation (Bayesian algorithm)	Environmental science
*Core II: “CO*_*2*_* price component for fossil fuels”*		
5. Impact of user cost increase for conventional vehicles of 2% p.a. on the vehicle market [n.a.]	Total-cost-of-ownership	Innovation economics
6. Law-making and monitoring options for CO_2_ price implementation [68]	Legal analysis	law
7. Impact monitoring for CO_2_ prices meeting the CO_2_-reduction pathway of 55% by 2030 [n.a.]	Simulation (system optimization model)	Energy economics
8. Impact of policy measures (e.g. CO_2_ price) on mobility behaviour and demand [69]	Simulation (agent-based model)	Behavioural economics
*Ancillary I: “Reform of the motor vehicle tax”*		
9. Impact of vehicle CO_2_ tax reform on total mobility costs and vehicle fleet distribution [n.a.]	End consumer cost calculation	Innovation economics
*Ancillary II: “Technology development for intelligent charging & tariff systems”*		
10. Analysis of capacity allocation options for integrating electromobility into the electricity system [n.a.]	Decision theory	Institutional economics
11. Analysis of various management models for implementing fast-charging electric vehicle infrastructure [n.a.]	Impact assessment	Institutional economics
12. Analysis of legal framework and problems for charging infrastructure [68]	Legal analysis	Law
13. Effectiveness of the use of intelligent charging points and vehicle fleet impact [n.a.]	Simulation (system optimization model)	Energy economics
*Ancillary III: “Guideline on parking fees”*		
14. Analysis of various options for public parking space regulation [n.a.]	Impact assessment	Institutional economics
15. Impact of parking fee increase on mobility behaviour patterns [n.a.]	Representative survey in two cities	Environmental psychology
*Ancillary IV: “Target group-oriented information campaign on electric mobility”*		
16. Impact of information campaigns pro electro mobility on different target groups [70]	Simulation (agent-based model)	Behavioural economics
17. Impact of information campaigns pro electro mobility among commercial customers [n.a.]	Interviews, survey (conjoint analysis)	Environmental psychology
18. Design of information campaign pro electro mobility for private households. [n.a.]	Survey (conjoint analysis)	Environmental psychology
19. Analysis of willingness to switch towards alternative drive cars [n.a.]	Representative survey in two cities	Environmental psychology

Source: own elaboration

*Indication of publication or non-publication as not available (n.a.) in square brackets. Summary results of non-published material is available from the authors on request

Several studies dealt with impact assessment of the *CO*_*2*_* emission performance standard.* Study no. 1 analysed the impact of CO_2_ limit values on the development over time of the fleet composition consisting of new vehicle registration and the remaining existing fleet. The work focused on re-assessing the current state of the art as described in [71]. As a result, the passenger car limits of 60 g/km in 2030 and 10 g/km in 2050 ensure both a significant increase in the efficiency of conventional passenger cars and an increasing proportion of electric vehicles. From a macroeconomic perspective, study no. 2 used a general equilibrium model to examine various scenarios, each of which used different policy measures, such as CO_2_ performance standard. The results indicate that new car purchases change the most over time in the “standard” scenario. In order to comply with the CO_2_ limits, there are both substitution effects between the demand classes and drive types, and budget effects in relation to the absolute level of new vehicle purchases. Further research (no. 3) comparatively analysed several instruments towards impact parameters, i.e. effectiveness, uncertainty (for vehicle suppliers), need for knowledge for different agents, need for commitment, revenue generation, and protection of specific investments. Finally, a value chain upstream analysis has been carried out (no. 4), assessing the risks of supply disruptions associated with vanadium-based redox flow batteries for the German market, using the Holistic Risk Analysis and Modelling (HoRAM) method.

The consequences of a *CO*_*2*_* price component* were analysed by four studies. From a microeconomic perspective, one piece of research (no. 5) used the total cost of ownership approach (TCO) to examine the specific effectiveness of the level of CO_2_ pricing proposed in the policy package. A law study no. 6 focussed on legal framework settings, and the potential and constraints for CO_2_ pricing implementation in Germany. A macroeconomic simulation study (no. 7) used the energy system model REMod in order to show the total avoidance costs arising in comparison with a business-as-usual scenario. As a target setting, the simulation was based on a total reduction of CO_2_ emissions of 55% by 2030 and deduced an adequate level of CO_2_ price to meet the reduction goal. In an agent-based model approach, study no. 8 depicted possible changes in mobility behaviour and mobility demand due to the increase in the cost of motorized private transport (MPT) via a CO_2_ price component.

The accompanying policy measure *reform of the motor vehicle tax* was covered by just one study. Research no. 9 dealt with the explicit design of the measurement and concluded that the three measures of CO_2_ price component, motor vehicle tax reform, and guidelines on parking space management altogether should lead to reduced motorized private transport patterns.

*Intelligent charging points and tariff systems* were the focus of four assessment studies. From an institutional economics perspective, analyses were carried out for researching capacity allocation options for integrating electro mobility into the electricity system (no. 10), and for researching various management models for implementing fast-charging electric vehicle infrastructure (no. 11). In addition, law analysis assessed the legal framework and problems for charging infrastructure (no. 12). From a techno-economic optimization approach, using the REMod simulation tool (no. 13), the effectiveness of intelligent charging stations use was analysed, and whether grid-supported charging and discharging of battery electric cars has an impact on their use in the vehicle fleet.

Two studies dealt with *guidelines on parking fees.* One study analysed various options for public parking space (no. 14), while study no. 15 carried out two representative surveys in big cities in order to assess the impact of a parking fee increase on mobility behaviour patterns.

Finally, the measure *target group-oriented information campaign on electric mobility* was assessed by four contributions. Study no. 16 used an agent-based model from innovation and diffusion research to investigate how a target-oriented information campaign would affect the diffusion of electric vehicles. Environmental psychology contributed two studies analysing the main information deficits that would have to be addressed by an information campaign (no. 17), and dealt with designing an information campaign pro electric mobility for private households (no. 18). Based on data available from the two city surveys, an analysis of the willingness to switch towards alternative drive cars, was carried out (no. 19).

#### The evaluation phase: synthesizing multicriteria impact profiles

The interdisciplinary impact assessment shown above provided a great variety of single results within the complex field of urban passenger transport in socio-technical systems. We developed a three-step approach for the evaluation of the impact assessment. First, we set up evaluation criteria according to different aspects of socio-technical systems and adapted them to the case of urban passenger transport. Second, we identified results from the impact studies assigning them to the criteria. Third, we evaluated qualitatively the single policy package measures according to the evaluation criteria.

Evaluation of impacts needs to rely on multidimensional criteria that cover the heterogeneity of socio-technical systems of humankind. We relied on a set of criteria proposed for the transformation of energy systems [64], which distinguishes five principal categories that address several dimensions of socio-technical systems and are equipped with corresponding criteria towards urban transport systems:

- *Technology development.* This includes criteria such as innovative mobility services, alternative drives for MPT, alternative drives for public transport, and intelligent charging infrastructure.
- *Sector integration and coupling.* This comprises the criteria of development of intelligent charging infrastructures, and coupling of renewable electricity generation with the energy demand in transport.
- *Environmental impact.* This includes traditional emissions (air, water, soil, noise), and greenhouse gases.
- *Social resonance.* This covers issues such as empirically measured willingness-to-accept (technologies, policy measures), and empirically measured consumption and investment behaviour (households, companies).
- *Institutional factors.* This includes legal barriers (contradictions, inefficiencies, etc.), political barriers (e.g. overlapping competencies, mismatches between vertical governance levels, lobbying, time delays, etc.), spatial barriers, and economic barriers.

We then identified relevant results from the individual impact studies according to categories and criteria. The aim was to specify relevant indications for the case study on urban transport across the multidimensional socio-technical systems categories from the individual impact studies. While relevant impact results were identified by hands of a survey among project groups, the assignment to categories and criteria was done by document analysis and bilateral conversations with the research teams. Table 3 gives an overview of the variables identified.

**Table 3:** Evaluation categories and criteria specified with results from the impact studies. Source: own elaboration based on [64]

*Criteria*	*Variables*
*Category I: Technology development*	
Innovative mobility services	• Use of electric vehicles for ride-sharing systems
Alternative drives for MPT	• Diffusion of electric vehicles in MPT (private and public) • Range development • Cost development
Alternative drives for public transport	• Diffusion of electric vehicles in public transport
Intelligent charging infrastructure	• Development and establishment of intelligent charging infrastructure to avoid system instability
*Category II: Sector integration*	
Intelligent charging infrastructure	• Avoidance of negative effects of the diffusion of electric vehicles on the electricity system through the development of charging possibilities that are beneficial to the system
Coupling of renewable electricity generation with the energy demand in transport	• Electrification of the transport sector only serves climate protection if the growing demand for electricity is met by the additional expansion of renewable energy supply • Electricity can be stored in battery electric vehicles directly at the time of generation, provided they are connected to the grid • Electricity can be used in electrolysis plants to produce hydrogen at the time of generation. This can be stored and used for refuelling independent of the electricity generation • Electrification thus serves the purpose of sector integration
*Category III: Environmental impact*	
Emissions to air, water, soil	• Production: mining and use of rare earths and critical resources (lithium, cobalt, platinum) may be problematic • Use phase: avoidance of NOx, reduction of fine dust, avoidance of further air pollutants in direct operation. Shift to electricity generation (if non-renewable energies are used)
Greenhouse gases	• Production: mining and use of rare earths and critical resources (lithium, cobalt, platinum) • Use phase: avoidance of CO_2_ emissions in direct operation, shift to electricity generation if non-renewable
*Category IV: Social resonance*	
Empirically measured willingness-to-accept	• Increase in the cost of private transport: intention to switch may be high, but there is a risk of social imbalance; alternatives (public transport and alternative drives) must be available and usable • Lack of information: there is a lack of neutral information and education about the technical characteristics and possibilities of alternative drives, which is why there is a great deal of scepticism about the new technologies
Empirically measured consumption and investment behaviour	• Purchase decision: depending on the level of information, the level of investment, the running costs, the technical characteristics such as range
*Category V: Institutional factors*	
Legal barriers	• Status quo: not everyone can participate equally (e.g. tenants cannot instal a charging infrastructure) • Lack of procurement guidelines: there are (still) no guidelines for public procurement to give preference to alternative drives
Political barriers	• Windows of opportunity: current problem pressure via EU specifications, society’s climate protection claim (Fridays for Future) • Lack of coordination: activities of the car industry, the energy sector and the state to establish charging infrastructures should be coordinated and more goal-oriented
Spatial barriers	• Contextual dependency: use of alternative drives, if necessary depending on the type of space (urban/rural), different incentives and systems of measures may be required
Economic barriers	• Investment costs: vehicles with alternative drive systems are sometimes significantly more expensive than conventional vehicles, lack of procurement guidelines

In the field of technology development, electric vehicles should not only be used in MPT, but also for innovative mobility service provision, and for public transport. Increased diffusion of alternative drive vehicles needs to be pushed and incentivized by policy measures. In parallel, development and installation of intelligent charging infrastructure needs to be encouraged in order to avoid energy system instability. In the area of sector integration, there is a need to avoid negative effects of the diffusion of electric vehicles on the electricity system at large. Thus, expansion of renewable energy sources is pre-requisite for the extended use of alternative drives. For better coupling between electricity generation and use, storage capacities can be used either with battery electric vehicle storage, or with hydrogen production. In the area of environmental impact, the production and use phases are likewise important. While raw material production for batteries is a crucial issue with considerable environmental impacts and uncertainties, alternative drives in the use phase assure improvements for both traditional emissions into air, water and soil, and reduced greenhouse gases.

Social resonance in the sense of social acceptance and social behaviour are of major importance as an evaluation category. As for alternative drives, there is an increase in the cost of private transport. Thus, willingness to switch may be high, but there is a risk of social imbalance. To compensate, alternatives like public transport must be available and affordable. In addition, there is a lack of neutral information and education about the technical characteristics and possibilities of alternative drives, causing a great deal of scepticism about the new technologies. Finally, in the field of institutional factors, several issues remain. Due to legal issues, not everyone can participate equally in using alternative drive vehicles (e.g. tenants cannot instal private charging infrastructure). As a political issue, there are windows of opportunity currently open for transitioning the transport system with problem pressure via EU specifications, and society’s climate protection claim (Fridays for Future). However, the current Covid-19 pandemic also favours private car driving in the existing (compulsion engine) vehicle fleet. In addition, there is a lack of coordination between activities of the car industry, the energy sector and the state to establish charging infrastructures. Regarding spatial issues, there are contextual dependencies with the use of alternative drives between urban and rural areas. Lastly, economic barriers exist towards investment costs. Vehicles with alternative drive systems are significantly more expensive than conventional vehicles. However, state subsidies for electric car purchase have been considerably extended.

### The discourse phase: iterative feedback loops with discursive dialogue

**Fig. 2:**
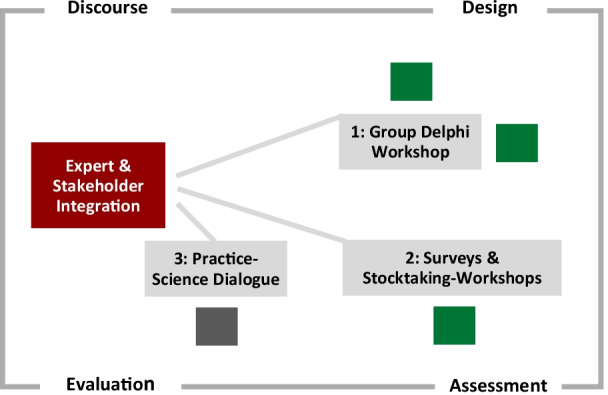
Spotlight on the discourse phase within the IPPA implementation. Source: own elaboration

The discourse phase applied several participatory approaches to discuss IPPA development and implementation with internal project staff and external stakeholders along several phases of the cycle. Figure 2 displays the participatory methods used at different stages of the IPPA implementation.

In the design phase, we applied a *Group Delphi Workshop.* A Group Delphi is a variant developed in the 1990s as a modification of the traditional Delphi method [72–76]. The Group Delphi Workshop discussed and assessed predefined target orientation, sustainable pathways identification and corresponding policy interventions and designed policy packages. It resulted in assessing levels of effectiveness, efficiencies and acceptance among adequate policy interventions, and corresponding trade-offs. In the case of the policy package for the “alternative drive systems” pathway, the expert assessments were very similar: firstly, infrastructure expansion has a major influence on all alternative drives discussed; secondly, there was ambivalence with regard to taxation and quantity limits; and thirdly, the accompanying measures were assessed as being very differentiable. The ambivalence regarding taxation and quantity limits was based on the following arguments. In favour of the CO_2_ tax is the fact that both existing users and those who want to purchase a new passenger car are affected. In addition, high effectiveness and low side-effects can be expected. The fact that taxes in the high price segment are a marginal aspect for buyers and accordingly have no steering effect on certain groups of buyers speaks against it. One argument in favour of quantity limits is that they are relatively easy to implement through regulation and sanctions, which manufacturers can use to adjust their internal price structure and ensure that the vehicles are purchased. However, there may be a consistency problem, as the quota must be combined with a corresponding infrastructure.

Focussed on the interdisciplinary impact assessment phase, we used the participative methods of *written surveys and stocktaking workshops*. The two-step survey approach gathered knowledge on impact studies carried out in the ENavi project and delivered specific details on background, leading research questions and methods used, summary and discussion of major results, and assignment to the corresponding measurements within the policy package. The Stocktaking Workshops introduced and discussed both the IPPA implementation procedure and the synthesis of impact assessments.

In the evaluation phase, we carried out a *Practice-Science Dialogue*. The policy package “alternative drives” (together with the policy package on “multi- and inter-modality”) was subject to critical examination from various perspectives. At this event, representatives from business, politics and civil society together with project staff discussed the preliminary IPPA results [77]. The workshop event aimed at the following objectives: to present the current research results from the IPPA approach, provide in-depth knowledge on selected individual impact study results, and discuss the overall approach of the IPPA. In presenting both pathway cases, we also stressed complementarity with firstly, encouraging shifts towards more efficient modes of passenger transportation, and secondly substitution towards alternative drives within the remaining fleet of vehicles. The recommendations derived from practitioners and stakeholders are depicted in Table 4. What became clear from the recommendations is that IPPA results were contextualized to a broader picture of mobility transition within a socio-technical system perspective.

**Table 4:** Practitioners’ and stakeholders’ recommendations from the practice–science dialogue

*Policy must offer visions: What does future climate-compatible mobility in rural and urban areas look like?*
For a successful transformation of transport with broad active support from society, we need above all a vision of what climate-friendly and sustainable transport will look like in 2030—in the city and in rural areas. Without visions, there can be no "I am in favour of it." Policymakers must drive the development of such visions and communicate them widely. They are the basis on which the transformation of transportation can be not only managed, but collectively shaped. In communicating these visions, openness about conflicting goals is needed; in implementing them, the courage is needed to take measures that are sensibly coordinated with one another, even if the final certainty that this is the perfect mix of measures is lacking. Now is a good time to put together packages of measures based on visions of sustainable mobility for cities and rural areas and to start implementing them in the short term. At the moment, there is an opportunity for people to look at traffic with a different lens. The Fridays for Future movement, with its demand for climate justice, as well as the defensive position in which the automotive industry—and the politics closely linked to it—find themselves as a result of the diesel scandal and the on-going fight against air pollution, are contributing to this opportunity
*Creating a socially just mobility transition*
The mobility transition must always be viewed from the perspective of a socially just mobility transition. Measures that "hit the wallet" are certainly considered promising. However, the decision-making bodies find themselves in a field of tension: while conflicts of interest must be balanced out in broadly conducted debates, these debates must not bring the decision-making process to a standstill. One possible measure to partially remedy this situation is to increase the transparency of the policymaking process in such a way that all actors are informed to the same extent and no information asymmetry arises between the various interest groups. For example, the planned use of the funds collected or the different design options for a pricing instrument should be clearly communicated. The use of pricing instruments requires the courage to engage in controversial debates and the ability to compromise, but must not disregard disadvantaged groups. Here, it helps to look beyond pricing instruments and consider other, restrictive measures that primarily affect the comfort of the car and less on the drivers' wallets. For example, an artificial parking shortage can lead to a significant reduction in the use of private cars and at the same time enhance public space, which in turn serves all population groups. If individual population groups are particularly strained financially by pricing instruments, it must be examined how social cushioning can be created here
*Creating suitable framework conditions through trial and error*
How we are mobile depends primarily on the existing infrastructure. Our environment is primarily characterized by MPT. Since infrastructure projects are usually cumbersome, lengthy and cost-intensive, it often makes sense to first test the effects of such measures scientifically and on site. For example, real-lab settings are suitable for this, especially when it comes to the redistribution of road space in favour of active mobility. Cycling and walking should generally be given more attention in the course of the mobility transition; unbureaucratic test sites can make a valuable contribution here on the way to a major reform of road traffic regulations. Federal policy must create a legal framework that gives municipalities the necessary leeway to reallocate urban areas to the detriment of MPT. The current road traffic regulations are primarily designed to ensure the safety and ease of car traffic. The deconstruction of car lanes and parking areas and the redesign of public spaces should take place in a participatory manner with the involvement of citizens and various stakeholder groups. In the promotion of active mobility, infrastructural measures must be supported by communicative measures, especially those that are linked to the testing of climate-friendly modes of transport or mobility spaces and can trigger "wow" effects. Opportunities for municipalities to try out different measures in an uncomplicated manner in terms of time and space thus also consider the specific nature of cities and our social spaces in general. In mobility, there are no one-size-fits-all solutions; experimental spaces make it possible to find the right solutions for the respective space
*Promotion of electric cars must be embedded in the transportation transition*
Switching passenger car propulsion from internal combustion to electric is one of the fastest ways to achieve climate targets. Therefore, measures for a stronger distribution of electric cars are necessary. At the same time, the so-called “drive turnaround” must be embedded in an overarching transport and mobility transition concept. This means that traffic must not only be improved, but also shifted to climate-friendly means of transport and avoided by shortening distances through sensible allocation of urban functions. To promote the spread of electric cars, manufacturer quotas for electric cars and other registration quotas (such as limits for combustion vehicles or weight limits for passenger cars) are preferable to purchase premiums. The latter have not proven to be effective levers in the past. Furthermore, there is a need for more choice in electric cars, uniform pricing models for charging stations so that charging costs become predictable, and information campaigns that dispel the myth of the range problem. If the electric car product makes cognitive and experiential sense to people, it will become (more) attractive even without a premium. The booming market for electric bicycles points to this

Source: own elaboration

## Discussion

In this paper, we presented an integrated approach for policy package assessment and illustrated the concept focusing on a case study in the area of urban passenger transport. Since many “grand challenges” are characterized by complexity, uncertainty, and ambiguity within socio-technical systems, integrated inter- and trans-disciplinary approaches seem promising—but they are much more difficult to apply. The IPPA approach consists of a four-phase framework model with design, assessment, evaluation and discourse policy packages. In the following, we discuss the main findings according to results on the levels of content and process perspective.

First, the scope and depth of content results from both the overall IPPA approach, and the individual impact assessment studies is promising. To begin with, the IPPA approach opens up the view of several crucial issues of today’s major challenges in problem-oriented science and policy: firstly, it shows interacting and embedded policies bundled in a package. Second, it considers a heterogeneity of impact perspectives yielding to very different but similarly important results. Thirdly, it provides an overall view on consistently evaluating the different results. And finally, it provides opportunities to discuss the integrated scientific results from real-world perspectives of practice experts from administration/policy, civil society, and business. Thus, the overall IPPA approach may serve as a materialized blueprint approach for analysing policy packaging as policy advice.

Second, the process of implementing the IPPA approach remains a major challenge. The conceptual framework of IPPA is an ideal-type approach which is difficult to fulfil in real-world science practice. The continuous need to compromise leaves room for inadequate results and thus for frustration. Challenges that arise during process implementation include: first, harmonizing timelines and time periods between those responsible in the process across the four process stages. Second, the definition and consideration of comparable policy measurement details within the impact studies are very challenging. These efforts are important in order to ensure all single studies have more-or-less the same research subject and yield comparable results. Finally, a discussion on how the core and ancillary policies relate directly and indirectly to specific impacts and what policy revisions are needed to increase effectiveness, efficiency and acceptance is essential.

## Conclusions

One may conclude that the IPPA approach is ambitious with considerable added-value for integrated science, but still has also some shortcomings from a content perspective. The accuracy match between policy package measurement details and consideration of exactly these specifications within the impact studies is difficult to reach. Thus, it was not always clear whether the impact studies adequately relied on the specific measurement configuration when carrying out their impact assessment. The inadequacy may be a result of insufficient consideration when designing the impact studies or a result of difficulties in translating the measurement details into the methods used (survey, simulation, etc.). Another shortcoming is the (non-)comparability or (in)commensurability of the single results. The heterogeneity of single results provision is an added-value for providing insights into real-world impact complexities, but simultaneously these are difficult to put into a coherent synthesis. Which results are more explanatory? Which are less relevant? This is difficult to assess. However, what still remains is the fact that integrated science approaches for policy advice seem to be the road to follow. Even if this road is a challenging one, the added-value of integrated approaches is early consideration of real-world complexities, uncertainties, and ambiguities. It will be worthwhile to spend future effort in trying to achieve solid and feasible concepts and practices.

## Data Availability

The authors confirm that the data supporting the findings of this study are available within the article and the data that support the findings of this study are available from the corresponding author, upon reasonable request.

## Footnotes

1 The research was conducted within the collaborative research project ENavi (“Kopernikus Navigation System for the Energy Transition” 2016–2019) [16–19]. The consortium gathered a wide range of technical, economic and social science expertise, with 59 associated partners and 26 associated experts who contributed their experience to the topics of infrastructure, heating and mobility. The project aim was to develop and implement an inter- and trans-disciplinary approach to integrate political interventions and measures, and work across the energy sectors of electricity, heat and mobility.
